# Comparative performance of pooled cohort equations and Framingham risk scores in cardiovascular disease risk classification in a slum setting in Nairobi Kenya

**DOI:** 10.1016/j.ijcha.2020.100521

**Published:** 2020-04-28

**Authors:** Frederick M. Wekesah, Martin K. Mutua, Daniel Boateng, Diederick E. Grobbee, Gershim Asiki, Catherine K. Kyobutungi, Kerstin Klipstein-Grobusch

**Affiliations:** a African Population and Health Research Center, Nairobi, Kenya; b Julius Global Health, Julius Center for Health Sciences and Primary Care, University Medical Center Utrecht, Utrecht University, the Netherlands; c Division of Epidemiology and Biostatistics, School of Public Health, Faculty of Health Sciences, University of the Witwatersrand, Johannesburg, South Africa; d Department of Global Health and Population, Harvard T.H. Chan School of Public Health, Harvard University, United States

**Keywords:** Risk, Risk assessment, Risk communication, Framingham, Pooled cohort equations, Kenya

## Abstract

**Background:**

Cardiovascular diseases (CVD) cause 18 million deaths annually. Low- and middle-income countries (LMICs) account for 80% of the CVD burden, and the burden is expected to grow in the region in the coming years. Screening for and identification of individuals at high risk for CVD in primary care settings can be accomplished using available CVD risk scores. However, few of these scores have been validated/recalibrated for use in sub-Saharan Africa (SSA).

**Methods:**

Pooled cohort equations (PCE) and Framingham risk scores for 10-year CVD risk were applied on 1960 men and women aged 40 years and older from the AWI-Gen (Africa, Wits-INDEPTH Partnership for GENomic studies) study 2015. Low, moderate/intermediate or high CVD risk classifications correspond to <10%, 10–20% and >20% chance of developing CVD in 10 years respectively. Agreement between the risk scores was assessed using kappa and correlation coefficients.

**Results:**

High CVD risk was 10.3% in PCE 2013, 0.4% in PCE 2018, 2.9% in Framingham and 3.6% in Framingham non-laboratory scores. Conversely, low CVD risk was 62.2% in PCE 2013 and 95.6% in PCE 2018, 84.0% and 80.1% in Framingham and Framingham non-laboratory scores, respectively. A moderate agreement existed between the Framingham functions (kappa = 0.64, 95% CI 0.59–0.68, correlation, r_s_ = 0.711). There was no agreement between the PCE 2013 and 2018 functions (kappa = 0.05, 95% CI 0.04–0.06).

**Conclusions:**

Newer cohort-based data is necessary to validate and recalibrate existing CVD risk scores in order to develop appropriate functions for use in SSA.

## 1 Introduction

Cardiovascular diseases (CVD) account for 17.9 million deaths annually, equivalent to 31% of all deaths globally [1], [2]. About 75% of these deaths, 85% of which are caused by heart attacks and stroke, occur in low- and middle-income countries (LMICs) [1]. Sub-Saharan Africa (SSA) contributes about 5.5% of global CVD deaths, the proportion estimated to double by 2030 [3]. Known risk factors for CVD include high systolic blood pressure, smoking, high fasting plasma glucose, high body-mass index, and particulate matter pollution [4].

The poor suffer a disproportionately higher burden of cardiovascular diseases. They are also affected by significant disparities in accessing health care and services [5]. In populations underserved by health care services, CVD risk is often perceived inappropriately: individuals display optimistic bias in their self-assessment for CVD risk (optimistic bias is when individuals think that they are less likely to develop CVD when compared with others) [6], [7]. Optimistic bias in individual risk assessment could be as result of the widespread low levels of awareness of CVD and risk factors, itself a result of the existing higher rates of illiteracy and widespread levels of social deprivation. Risk misperception results in late diagnosis and detection of CVD, leading to higher rates of premature deaths: deaths occurring among individuals aged below 70 years.

Assessing absolute cardiovascular risk is a proven clinically sound guide to prevent and promote adherence to treatment strategies for CVD [8], [9]. It is a useful tool in risk communication for primary prevention [10]. By calculating a patient’s absolute risk for CVD, a health care provider can identify individuals at increased risk for CVD and recommend mitigation strategies [11], [12], [13]. Absolute risk assessments and communication to the affected individuals have been used in raising CVD awareness, and in motivating adherence to lifestyle modifications and/or treatment in the developed world [14].

A number of multivariable CVD risk assessment algorithms (risk scores) that incorporate known and novel risk factors for CVD have been developed and are in use globally [15], [16], [17], [18], [19], [20], [21], [22], [23]. Majority of these risk scores were however developed in predominantly Caucasian populations, and may not be applicable to other populations, especially African. For instance, an earlier analysis reported that the Framingham-based scores had poor calibration when applied to certain ethnic groups and socioeconomically deprived populations in underestimating risk [24].

Few of the CVD risk scores are modified for use in screening populations in SSA. The current study assesses the performance of the newly released 2018 Pooled Cohort Equations (PCE) risk score for atherosclerotic cardiovascular diseases (ASCVD) as compared to the original (2013) PCE and the Framingham risk scores (laboratory- and non-laboratory based) for CVD risk stratification in an urban low resource population. We aimed to assess the level of agreement between and among the functions, and their transportability and potential application in CVD risk assessment in under-resourced settings.

## 2 Data sources

We used data collected in 2015 from the AWI-Gen (Africa, Wits-INDEPTH Partnership for GENomic studies) study in the Nairobi Urban Health and Demographic Surveillance System (NUHDSS). The study involved 2003 participants (922 (46.03%) male) aged between 35 and 67 years old. The AWI-Gen study investigates the genetic, genomic and environmental risk factors associated with obesity and CVD in African populations [25]. The study is concurrently conducted in five other rural and urban demographic surveillance sites in Burkina Faso (Nanoro), Ghana (Navrongo), and South Africa (Agincourt, Dikgale and Soweto). For the current analysis forty-three individuals with incomplete records were excluded. This number included one individual diagnosed with stroke and 42 individuals with missing data on the key variables useful for the current analysis (i.e. blood glucose, blood cholesterol and blood pressure values, diagnosis and treatment for diabetes and hypertension and height and weight values).

## 3 Definitions

### 3.1 Pooled cohort equations

Pooled cohort equations were developed in the United States for CVD risk assessment and to address concerns with existing risk scores for being non-representative or developed from older cohorts, of limited ethnic diversity, and accommodating narrowly defined endpoints (usually coronary heart disease). The 2013 PCE were developed to estimate first hard atherosclerotic cardiovascular disease (ASCVD) events for endpoints that included CHD death, nonfatal myocardial infarction, and fatal or nonfatal stroke [26].

Race- and sex-specific PCE were recommended for use in non-Hispanic African Americans and non-Hispanic whites, 40 to 79 years of age. The 2013 PCE have been criticized for overestimating risk by between 20% and 150% across risk groups and in different populations [27]. Overestimation of risk was particularly a problem with the black race [28]. The 2018 PCE were developed to address this criticism. To derive the revised PCE function the same set of factors like the 2013 PCE (shown in Table 1) was used applying newer data and novel statistical methodology. Updating these equations is said to have improved accuracy among the race and sex subgroups and reduced the number of persons considered to be at high risk [28].

**Table 1:** Factors and cardiovascular outcomes for pooled cohort equations to assess 10-year ASCVD risk and Framingham risk functions for 10-year CHD outcomes.

	2013 Pooled cohort equations	2018 Pooled cohort equations	Framingham laboratory score	Framingham non-laboratory score
Predictors	Age, total cholesterol, HDL-cholesterol, systolic blood pressure, (treated or untreated), diabetes, and current smoking status	Age, total cholesterol, HDL-cholesterol, systolic blood pressure, (treated or untreated), diabetes, race, and current smoking status	Age, systolic blood pressure, anti-hypertensive medication use, current smoking, diabetes, *HDL-cholesterol*	Age, systolic blood pressure, anti-hypertensive medication use, current smoking and diabetes, *body-mass index*
Age group	40 – 79	40 – 79	30 – 75	30 – 75
Cardiovascular outcomes	Nonfatal myocardial infarction (MI), death from coronary heart disease, or fatal or nonfatal stroke	Nonfatal myocardial infarction (MI), death from coronary heart disease, or fatal or nonfatal stroke	Coronary heart disease, cerebrovascular, and peripheral artery disease and heart failure	Coronary heart disease, cerebrovascular, and peripheral artery disease and heart failure
Estimates	Sex-and race-specific	Sex-specific	Sex-specific	Sex-specific

### 3.2 The Framingham cardiovascular disease risk score

The Framingham cardiovascular risk score (FRS) [19], [29] is perhaps the best known and widely used function in CVD risk assessment globally. This sex-specific risk score is used to predict the occurrence of coronary heart disease events, as well as cerebrovascular, and peripheral artery disease and heart failure events within 10 years of baseline risk assessment [19]. The main criticism of the FRS (Framingham Wilson 1998), however, is that since it was developed predominantly in a white middle-aged population, it may not be applicable to racially/ethnically diverse and elderly populations [24]. The function has nevertheless been adapted, recalibrated and validated for use in many settings globally [30], [31]. The non-laboratory Framingham risk score replaces HDL-Cholesterol with body mass index. When checked against the laboratory-based score, it performed reasonably well and was recommended for use in under-resourced settings where laboratory tests may be unavailable or expensive to carry out [19].

### 3.3 Other measurements and definitions

Raised blood pressure was defined as systolic blood pressure (SBP) ≥ 140 mmHg and/or diastolic blood pressure (DBP) ≥ 90 mmHg. Hypertension was based on self-report of a previous diagnosis by a clinician, and/or current use of antihypertensive medication. Blood pressure (BP) was measured using an automated digital blood pressure device (OMRON™). Using appropriate cuff sizes for each individual, three readings were taken on the left arm from an individual in a seated position, at one minute intervals. The mean of the second and third measurements were used for the current analysis.

Raised blood glucose was classified based on plasma glucose concentration of ≥ 11.1 mmol/L or fasting plasma glucose of 7.0 mmol/L. A drop of blood from a finger prick was used to test for glucose using the ACCU-CHEK™ Glucose, Cholesterol and Triglycerides (GCT) digital meter. Diabetes was based on self-report of a previous diagnosis by a clinician and or current use of medication. A drop of blood from a finger prick was used to test for blood cholesterol using ACCU-CHEK™ GCT. Total blood cholesterol levels were categorized either as ideal or high (cut-off 5.2 mmol/L). Current cigarette smoking, previous diagnosis with stroke and/or heart attack were self-reported. Height was measured in centimeters using SECA™ height boards while the individual stood on a flat surface. Body weight was taken in kilograms using calibrated SECA™ weighing scales.

## 4 Analysis

Pooled cohort risk equations for Africans (2013 and 2018) and the Framingham risk scores (laboratory and non-laboratory) were applied and for each individual a risk score for developing ASCVD and CHD within a 10-years following the baseline risk assessment was computed. Cardiovascular risk was categorized into low (<10%) moderate/intermediate (10–20%) and high (>20%) respectively.

The agreement between any two functions was measured using a kappa-statistic, with the scores interpreted as guided by McHugh [32]: no agreement (0–20); minimal agreement (0.21–0.39); weak agreement (0.40–0.59); moderate agreement (0.60–0.79); strong agreement (0.80 – 0.90); and almost perfect agreement (0.91–1.00). Correlation between any two functions was assessed using Spearman’s correlation co-efficient. The Spearman correlation coefficient, r_s_, can take values from −1 to +1, with +1 indicating a perfect association, 0 indicating no association an −1 indicating a perfect negative association. The closer the r_s_ values are closer to zero, the weaker the association.

## 5 Ethical considerations

The AWI-Gen Kenya study received ethics approval from the Ethics and Scientific Review Committee of Amref (Ref #P114/2014). Individual written informed consent was sought from all participants who were informed that their participation in the study was voluntary and they could discontinue their participation in the study whenever they chose to. Participants diagnosed with raised blood pressure/glucose were referred for care.

## 6 Results

A total of 1960 records were included in the analysis. Men (mean age 49.19 ± 6.02 years) were slightly older than women (48.51 ± 5.59 years). More men (91.0%) were in a marital union i.e. living together, cohabiting or married compared to the women (45.7%). Fewer men (4.0%) compared to women (10.9%) possessed no formal education, while more men (45.0%) than women (26.2%) possessed secondary school level education or higher. Still fewer men (3.1%) compared to women (8.4%) were unemployed, while self-employment was more prevalent among women (58.8% vs 34.1%). More women (30.9% & 32.4%) compared to men (19.3% & 5.6%) were overweight and obese. Underweight was three times more common in men (11.6%) compared to women (3.8%). Tobacco use and smoking was prevalent among men (23.7%) compared to 2.6% among women. Overall, there were sex-differences on each key factor except on the high-density lipoprotein (HDL)-cholesterol levels, mean diastolic blood pressure (DBP), overall raised blood pressure levels, and on the proportion currently or previously on treatment for hypertension. Other characteristics of the study population are summarized in Table 2.

**Table 2:** Characteristics of the study population (N = 1960).

Factor	Overall (N = 1960)	Women n = 1060	Men n = 900
Age (years) Mean (SD)	48.8 (5.8)	48.5 (5.6)	49.2 (6.0)
Total cholesterol (mg/ml), Mean (SD)	167.1 (41.2)	170.4 (41.3)	163.1 (40.8)
SBP (mean mmHg), Mean (SD)	120.16 (21.0)	117.9 (21.6)	122.6 (20.0)
DBP (mean mmHg), Mean (SD)	78.3 (12.7)	78.4 (13.2)	78.3 (12.1)
HDL (mg/ml), Mean (SD)	48.9 (18.1)	48.5 (17.1)	49.3 (19.2)
Education			
No schooling n (%)	151 (7.7)	115 (10.9)	36 (4.0)
Primary school education n (%)	1126 (57.5)	667 (62.9)	459 (51.0)
Secondary school education and higher n (%)	683 (34.9)	278 (26.2)	405 (45.0)
Marriage			
In marital union n (%)	1303 (66.5)	484 (45.7)	819 (91.0)
Not in marital union n (%)	657 (33.5)	576 (54.3)	81 (9.0)
Occupation			
Self-employed n (%)	930 (47.5)	623 (58.8)	307 (34.1)
Formal employment n (%)	305 (15.6)	67 (6.3)	238 (26.4)
Informal (casual) employment n (%)	608 (31.0)	281 (26.5)	327 (36.3)
Unemployed n (%)	117 (6.0)	89 (8.4)	28 (3.1)
Current smoker n (%)	241 (12.3)	28 (2.6)	213 (23.7)
Blood pressure (140/90 mmHg) n (%)	389 (19.9)	208 (19.6)	181 (20.1)
Diagnosed with hypertension n (%)	319 (16.3)	231 (21.8)	88 (9.8)
Treatment for hypertension n (%)	251 (78.7)	190 (82.3)	61 (69.3)
Diagnosed with diabetes (Yes) n (%)	62 (3.2)	44 (4.2)	18 (2.0)
Treatment for diabetes (Yes) n (%)	48 (77.4)	33 (75.0)	15 (83.3)
Body mass index (Kg/m^2^)			
Underweight (<18.5 ) n (%)	145 (7.4)	41 (3.9)	104 (11.6)
Normal weight (18.5 – 24.9 ) n (%)	920 (46.9)	348 (32.8)	572 (63.6)
Overweight (25 - <30 ) n (%)	502 (25.6)	328 (30.9)	174 (19.3)
Obesity (≥30 ) n (%)	393 (20.1)	343 (32.4)	50 (5.6)

### 6.1 Classification of 10-year risk for CVD morbidity/mortality

**Fig. 1:**
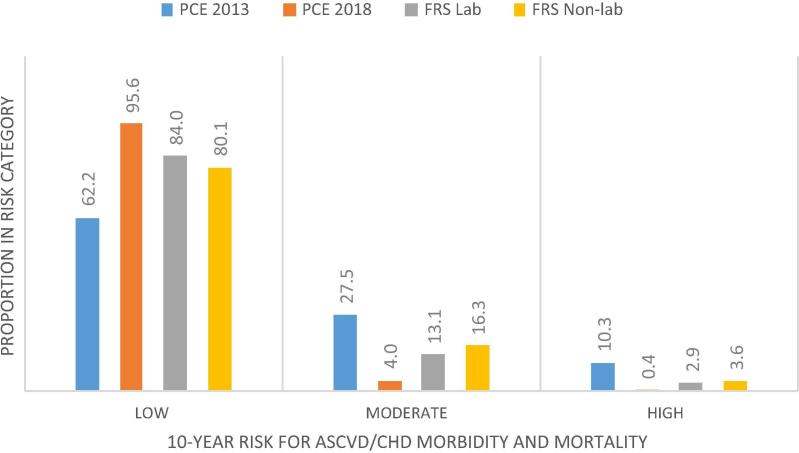
CVD risk classification of population in low resource settings in Nairobi.

Comparative classification of 10-year CVD risk by the PCE and Framingham risk functions is shown in Fig. 1. The proportion of individuals classified in the high risk group (>20% probability of developing CVD in 10 years) were highest (10.3%) in PCE 2013, and lowest (0.4%) in the PCE 2018; was 2.9% in Framingham and 3.6% in Framingham non-laboratory scores. Low risk category (<10% probability of developing CVD in 10 years) was lowest (62.2%) in PCE 2013, and highest (95.6%) in PCE 2018. The proportion of low risk was 84.0% and 80.1% in Framingham and Framingham non-laboratory scores, respectively.

### 6.2 Comparative risk classification

We checked the agreement and the correlation between any two of the four functions when applied in this population. The PCE 2013 when compared to PCE 2018, the kappa statistic (kappa) was lowest at 0.05 (95% CI 0.04–0.06), indicating no agreement between the scores. The Spearman’s correlation, r_s_, was also low at 0.39. There was a moderate agreement and a better correlation between the pair of Framingham functions (kappa = 0.64, 95% CI 0.59–0.68; r_s_ = 0.71). The agreement between the revised 2018 PCE and Framingham laboratory function was low (kappa = 0.29, 95% CI 0.24–0.34), as well as the correlation (r_s_ = 0.46). Likewise, there was minimal agreement and correlation between PCE 2018 and Framingham non-laboratory functions (kappa = 0.25, 95% CI 0.20–0.30; r_s_ = 0.41). Detailed information on the agreement and correlation in the functions is shown in Table 3.

**Table 3:** Agreement and correlation in cardiovascular risk classification among Framingham risk scores and pooled cohort equations.

		Pooled cohort equations 2013				Framingham laboratory				Framingham non-laboratory			
		Low n (%)	Intermediate n (%)	High n (%)	Total	Low n (%)	Intermediate n (%)	High n (%)	Total	Low n (%)	Intermediate n (%)	High n (%)	Total
Pooled cohort equations 2018	Low	1220 (65.1)	0 (0.0)	0	1220	1639 (99.6)	209 (81.3)	26 (45.6)	1874	1563 (99.6)	266 (83.1)	45 (63.4)	1874
	Intermediate	537 (28.7)	1 (1.3)	0	538	7 (0.4)	48 (18.7)	24 (42.1)	79	6 (0.4)	54 (16.9)	19 (26.8)	79
	High	117 (6.2)	78 (98.7)	7 (100.0)	202	0 (0.0)	0 (0.0)	7 (12.3)	7	0 (0.0)	0 (0.0)	7 (9.8)	7
	Total	1874	79	7	1960	1646	257	57	1960	1569	320	71	1960
	Kappa (95% CI)	0.05 (0.04–0.06)				0.292 (0.24–0.34)				0.248 (0.20–0.30)			
	p-value	<0.001				<0.001				<0.001			
	Spearman's r_s_	0.39**				0.46**				0.41**			
Framingham non-laboratory	Low	1104 (70.4)	115 (35.9)	1 (1.4)	1220	1517 (96.7)	125 (39.1)	4 (5.6)	1646				
	Intermediate	410 (26.1)	114 (35.6)	14 (19.7)	538	51 (3.3)	182 (56.9)	24 (33.8)	257				
	High	55 (3.5)	91 (28.4)	56 (78.9)	202	1 (0.1)	13 (4.1)	43 (60.6)	57				
	Total	1569	320	71	1960	1569	320	71	1960				
	Kappa (95% CI)	0.23 (0.20–0.30)				0.64 (0.59–0.68)							
	p-value	<0.001				<0.001							
	Spearman's r_s_	0.41**				0.71**							
Framingham laboratory	Low	1189 (72.2)	31 (12.1)	0 (0.0)	1220								
	Intermediate	404 (24.5)	129 (50.2)	5 (8.8)	538								
	High	53 (3.2)	97 (37.7)	52 (91.2)	202								
	Total	1646	257	57	1960								
	Kappa (95% CI)	0.31 (0.28–0.35)											
	p-value	<0.001											
	Spearman's r_s_	0.54**											

Key: *Kappa statistic*: a measure of agreement between a pair of items across the different levels. Ranges from 0 (agreement equivalent to chance) to 1 (perfect agreement); *Spearman’s correlation* r_s_: a statistical measure of strength and direction of association between two ordinal/ranked variables. + 1 indicating a perfect association, 0 indicating no association an −1 indicating a perfect negative association.

## 7 Discussion

The proportion of the population classified as high risk (>20% probability of developing ASCVD/CHD within 10-years of risk assessment) ranged from a low of 0.4% to a high of 10.3% while it ranged from 4.0% to 27.5% for the moderate/intermediate risk classification. Overall, a larger proportion of the population (range from 62.0% to 95.0%) were classified as low risk (<10% probability of developing ASCVD/CHD within 10-years of risk assessment).

When compared with the 2018 PCE, the 2013 PCE classified relatively more individuals into the high and moderate/intermediate risk categories. This tendency of the 2013 PCE to ‘over-classify’ was highlighted by the developers of the 2018 PCE functions when they set out to revise them [28]. The current findings on risk classification seem consistent with this attribute of overestimation for intermediate and high risk categories by 2013 PCE. Consistently therefore, if used in clinical practice in these settings, the PCE 2013 has the potential to misclassify individuals into high or in intermediate risk groups who may not be, and by so doing erroneously adding to the number of individuals needed to treat for CVD risk as high risk candidates [12], or even worse, needlessly alarming persons with low risk for CVD [19].

Our findings did not observe any substantial/strong agreement or correlation between any pair among the four functions considered in this analysis to warrant substitution in their use for screening in this urban slum community. The ‘best of the rest’ agreement was recorded between the pair og Framingham functions, indicating a moderate agreement with a positive but moderate correlation, while no agreement was seen between the pair of PCE functions.

The performance of the PCE and the Framingham functions has been investigated mostly in high income countries and in Asia [33], [34], [35]. However, Boateng et al [36] recently compared PCE 2013 and Framingham laboratory and non-laboratory functions among rural and urban Ghanaians in Ghana, and migrant Ghanaians living in Europe and the United Kingdom. In that study, PCE and Framingham non-laboratory scores posted better agreement in Ghanaian populations in Ghana as opposed to those residing in Europe and the UK. In China, the results of a comparative performance between PCE 2013 and Framingham risk scores showed substantial agreement between Framingham non-laboratory and Framingham laboratory functions at levels similar to our own findings, but there was a moderate agreement between PCE and Framingham laboratory, and a fair agreement between PCE 2013 and Framingham non-laboratory [34].

A comparison of the FRS laboratory and the 2013 PCE in the incidence of metabolic syndrome in a Korean population reported a 1.7 times (70%) increase (overestimation) in the high risk group by the PCE function [35]. These three studies demonstrate that the performance of existing CVD risk functions may be influenced by the population differences and contexts in which they are applied [37]. It is true, therefore, that CVD risk functions developed in and for specific population groups will misestimate (overestimate or underestimate) risk when used on other populations, evidently due to different baseline risks, owing to secular, cultural, contextual and epigenetic differences [38], [39].

Since the Framingham non-laboratory function was developed as an alternative to the laboratory function and was proposed as an alternative for use in resource-constrained settings where laboratory tests may be unavailable or expensive to carry out, anything less than an almost perfect agreement in their performance makes their use very limited. Transportability and use of functions in populations in which they were not created is evidently therefore a problematic matter. Each context may require its own function developed by recalibrating the existing functions by adding context-specific variables to the ones specified in the original functions [40]. This approach in recalibrating existing functions is less costly, and can improve the reclassification of individuals at intermediate risk as either being above or below a chosen intervention threshold.

A more robust approach, however, in developing appropriate context-specific risk scores is use of prospective data e.g. from cohort studies with a longer follow-up. Such cohort data is missing in SSA majorly due to the costs of setting up prospective studies, follow-up and ascertainment of outcomes. If available, cohort data can support the development of simpler non-laboratory measures with similar sensitivity and specificity when compared to the laboratory measures for use in primary health care settings. For SSA, CVD risk scores can incorporate socioeconomic variables like socioeconomic status and education which have been linked to CVD morbidity and mortality [43].

Our analysis faces some limitations. Data used in this analysis was from a cross-sectional study conducted in an urban slum community, and findings may therefore not be generalizable to the general population in Kenya. Without outcome data on fatal and non-fatal CVD events to validate the observed from expected outcomes, this study cannot comment on the appropriateness of any of the four functions for use in these settings. Our aim was limited to demonstrating their comparative performance in risk stratification in an underserved African population, and to lend a voice to the opinion on transportability and applicability of existing CVD risk functions across diverse populations.

The absolute risk approach can promote CVD prevention and enhance adherence to treatment when accompanied by effective risk communication in high risk individuals. Thus, beyond the development of appropriate risk functions, it is pertinent that they are disseminated to health care providers in primary health care settings, who should be sensitized about their general use in assessing for cardiovascular risk primary health facilities. Emphasis should be laid on the benefits of risk stratification and effective communication that go beyond the identification of individuals at high-risk, but encompasses the motivation for and promotion of adherence to risk mitigation [14]. Combining risk assessment with innovative approaches like the use of community health workers to screen, identify and follow up high risk individuals [41] and use of mobile phone health technology to promote messages to motivate risk mitigation [42] can deliver impressive results in CVD risk prevention in under-resourced settings, and can help to lower the incidence and the burden of CVD [14].

## Funding

FMW received the Global Health Support Award for PhD from University Medical Center, Utrecht. The Awigen Collaborative Center is funded by the 10.13039/100000051National Human Genome Research Institute (NHGRI), the 10.13039/100009633Eunice Kennedy Shriver National Institute of Child Health and Human Development (NICHD) and Office of the Director (OD) of the 10.13039/100000002National Institutes of Health (NIH) of the United States of America (Grant #U54HG006938), as part of the H3Africa Consortium.

## CRediT authorship contribution statement

FMW, DEG, CKK & KKG conceptualized the study and drafted the plan of analysis. FMW conducted the analysis supported by MKK and DB, while GA advised on and supported the drafting of the manuscript. KKG reviewed and gave feedback on the analysis and the manuscript drafts at all stages of preparation. All authors reviewed and provided substantial input into the manuscript.

## Declaration of Competing Interest

The authors declare that they have no known competing financial interests or personal relationships that could have appeared to influence the work reported in this paper.
